# Supplementary material for: Complexity of Genomic Epidemiology of Carbapenem-Resistant Klebsiella pneumoniae Isolates in Colombia Urges the Reinforcement of Whole Genome Sequencing-Based Surveillance Programs
Source: Clin Infect Dis. 2021 Nov 25;73(Suppl 4):S290–9. doi: 10.1093/cid/ciab777 (PMC8634422; doi:10.1093/cid/ciab777)
Supplement: ciab777_suppl_Supplementary_Material [file ciab777_suppl_supplementary_material.docx]

**Supplementary Information**

**Complexity of Genomic Epidemiology of Carbapenem-Resistant *Klebsiella pneumoniae* Isolates in Colombia Urges the Reinforcement of Whole Genome Sequencing-Based Surveillance Programs**

Sandra Yamile Saavedra^1^, Johan Fabian Bernal ^2^, Efraín Montilla-Escudero^1^, Stefany Alejandra Arévalo ^2^, Diego Andrés Prada^3^, María Fernanda Valencia^2^, Jaime Enrique Moreno^3^, Ángela Sofía García-Vega^2^, Andrea Melissa Hidalgo^1^, Monica Abrudan^4^, Silvia Argimón^4^, Mihir Kekre^4^, Anthony Underwood ^4^, David M Aanensen^4^, Carolina Duarte ^1^ and Pilar Donado-Godoy^2**^ and the NIHR Global Health Research Unit on Genomic Surveillance of Antimicrobial Resistance^a^

^1^ Grupo de Microbiología, Instituto Nacional de Salud (INS), Bogotá, Colombia

^2^ Colombian Integrated Program for Antimicrobial Resistance Surveillance – Coipars, CI Tibaitatá, Corporación Colombiana de Investigación Agropecuaria (AGROSAVIA), Tibaitatá – Mosquera, Cundinamarca, Colombia

^3^ Grupo de Microbiología, Dirección de Investigación en Salud Pública, Instituto Nacional de Salud, Bogotá, Colombia.

^4^ Center for Genomic Pathogen Surveillance – NIHR Global Health Research Unit (GHRU). Wellcome Sanger Institute. United Kingdom

^a^ Members of the NIHR Global Health Research Unit on Genomic Surveillance of Antimicrobial Resistance are listed in the Acknowledgments.

** Corresponding author

**SUPPLEMENTARY METHODS**

**Description of Colombian AMR Surveillance: Antimicrobial Resistance Program in Health Care-Associated Infections (*Infecciones Asociadas a la Atención en Salud* IAAS)**

The AMR surveillance by the laboratory in healthcare-associated infections (HAI) modifies the criteria for isolates submission according to the epidemiological behavior observed in the country and the epidemiological alerts from the Pan American Health Organization (PAHO) / World Health Organization (WHO).

For 2013, isolates resistant to at least one 3rd-generation cephalosporin with resistance or decreased susceptibility to at least one carbapenem were received following the guidelines 057 of 2012 (<https://www.ins.gov.co/Normatividad/Circulares/CIRCULAR%200057%20DE%202012.pdf#search=circular%20057>), and 043 of 2013 (<https://www.ins.gov.co/Normatividad/Circulares/CIRCULAR%20EXTERNA%200021%20DE%202014.pdf#search=circular%20021%20de%202014>).

For 2014 and 2015, the referral was carried out according to guideline 021 of 2014, meaning: i) isolates resistant to at least one 3rd-generation cephalosporin and with resistance or decreased susceptibility to at least one carbapenem; ii) positive results to phenotypic ethylenediaminetetraacetic acid (EDTA)/sodium mercaptoacetic acid (SMA) test; and iii) the first isolation of carbapenemase-producing enterobacteria from each institution after screening with modified Hodge test (MHT) and synergy with boronic acid (APB) disc test.

In 2016, a delivery criteria flowchart was established, and this is in force to date (<https://www.ins.gov.co/buscador-eventos/Informacin%20de%20laboratorio/Criterios-para-env%C3%ADo-de-aislamientos-bacterianos-y-levaduras-del-g%C3%A9nero-C%C3%A1ndida-en-IAAS.pdf> ). In general, the same criteria for the submission of isolates established for 2014-2015 were maintained, while isolates with a negative result to EDTA / SMA and APB test were included.

**Sample Selection**

From 2013 to 2017, the NRL received 811 clinical isolates of *K. pneumoniae* non-susceptible to carbapenems. Only one representative isolate of clinically defined outbreaks was included. Upon reception, bacterial identification was performed using ≈ 2 (bioMérieux), antimicrobial susceptibility was determined by disk diffusion, and results were interpreted using CLSI guidelines for the corresponding year. Phenotypic detection of carbapenemases was performed with Hodge test, EDTA test and acid 3-aminophenylboronic test (APB), while molecular detection was carried out using PCR for any positive CRKP, following Ovalle et al [1] to confirm the carbapenemase presence. However, the PCR result was not considered an exclusion criterion.

Out of the 811 confirmed as carbapenems resistance isolates, 557 were selected to be sequenced, the isolates were recovered on MacConkey agar, and their species and susceptibility to carbapenems were re-confirmed with VITEK 2 (bioMérieux), following the interpretation breakpoints from CLSI 2020. Of these, 132 samples were subsequently excluded, either because the isolates became nonviable, were identified as a species other than *K. pneumoniae,* or were not confirmed as CRKP.

**Classification of medical services by complexity**

The level of complexity for medical services was defined and reported by each institution according to the National Guideline 5261 of 1994 (<https://www.minsalud.gov.co/Normatividad_Nuevo/RESOLUCI%C3%93N%205261%20DE%201994.pdf>) and the Technical Annex of the National Guideline 1043 of 2006 on the Ministry of Health (<https://www.ins.gov.co/TyS/programas-de-calidad/Documentos%20Programa%20EEDDCARIO/resolucion%201043%202006.pdf>), which established the levels of complexity according to the institution installed capacity, specialization degree of medical personnel and, level of surgical services provided.

Medium complexity: describes institutions with basic specialties such as pediatrics, general surgery, internal medicine, orthopedics, and gynecology with 24-hour availability in hospitalization and emergency assessment; they also offer external consultation services by specialists and laboratories of greater complexity. Requires physician professionals.

Medium – high complexity: refers to institutions with an intermediate level of complexity since they provide medium and high-complexity medical services simultaneously.

High complexity: refers to institutions that have highly complex services that include specialties such as neurosurgery, vascular surgery, pulmonology, nephrology, dermatology, and others, with 24-hour specialist care, consultation, emergency service, interventional radiology, nuclear medicine, special units such as intensive care and a renal unit. Requires specialist professionals together with physcicians.

**Supplementary Tables**

**Supplementary Table 1.** Detailed quality control (QC) results for all sequenced isolates submitted as *Klebsiella pneumoniae*.

[Provided as Excel spreadsheet]

**Supplementary Table 2.** Demographic, phenotypic and genotypic characteristics of 425 isolates of carbapenem-resistant *Klebsiella pneumoniae*.

[Provided as Excel spreadsheet]

**Supplementary Table 3.** Correlation CG_ST_Department Mechanism resistance to carbapenem K-locus and *wzi* O-locus virulence factor in the clonal group and singletons.

[Provided as Excel spreadsheet]

**Supplementary Figures**


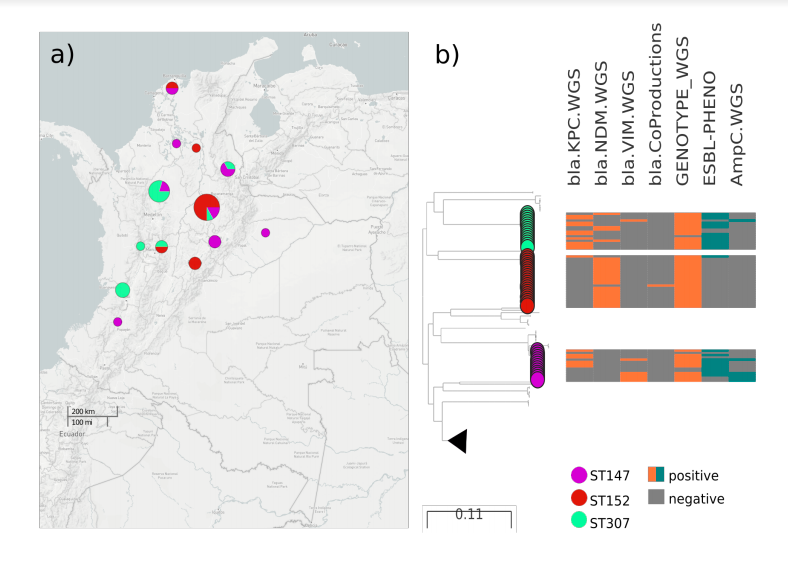


**Supplementary Figure 1.** Geographic and phylogenetic distribution of ST147, ST152, and ST307.


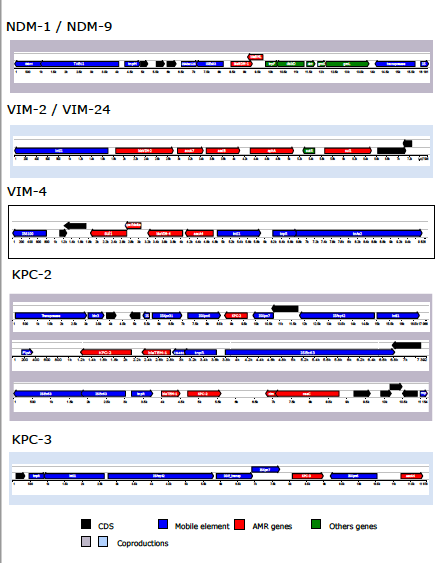


**Supplementary Figure 2.** Carbapenemase genetic environments. (Available as pdf.)


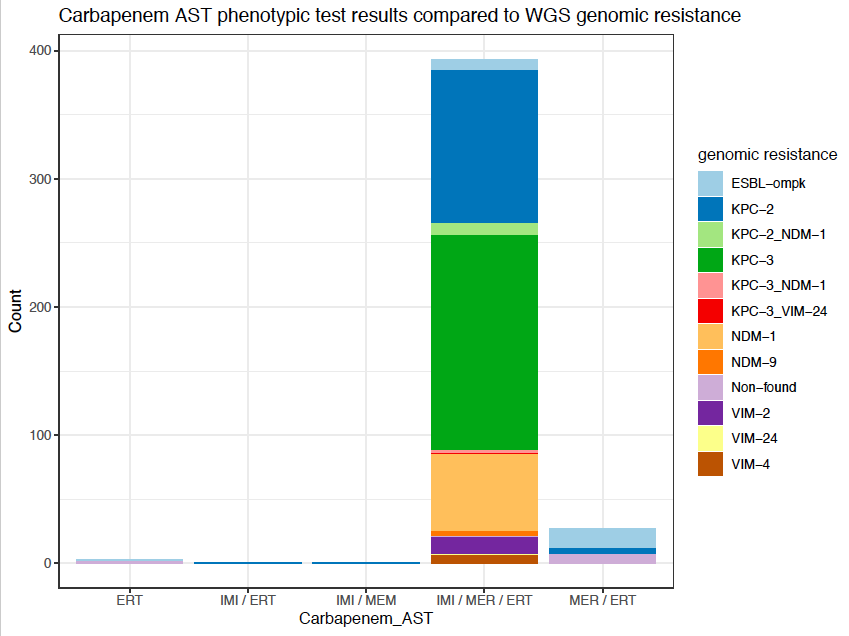


**Supplementary Figure 3.** Carbapenem AST phenotypic test results compared to WGS genomic resistance. (Available as pdf.)


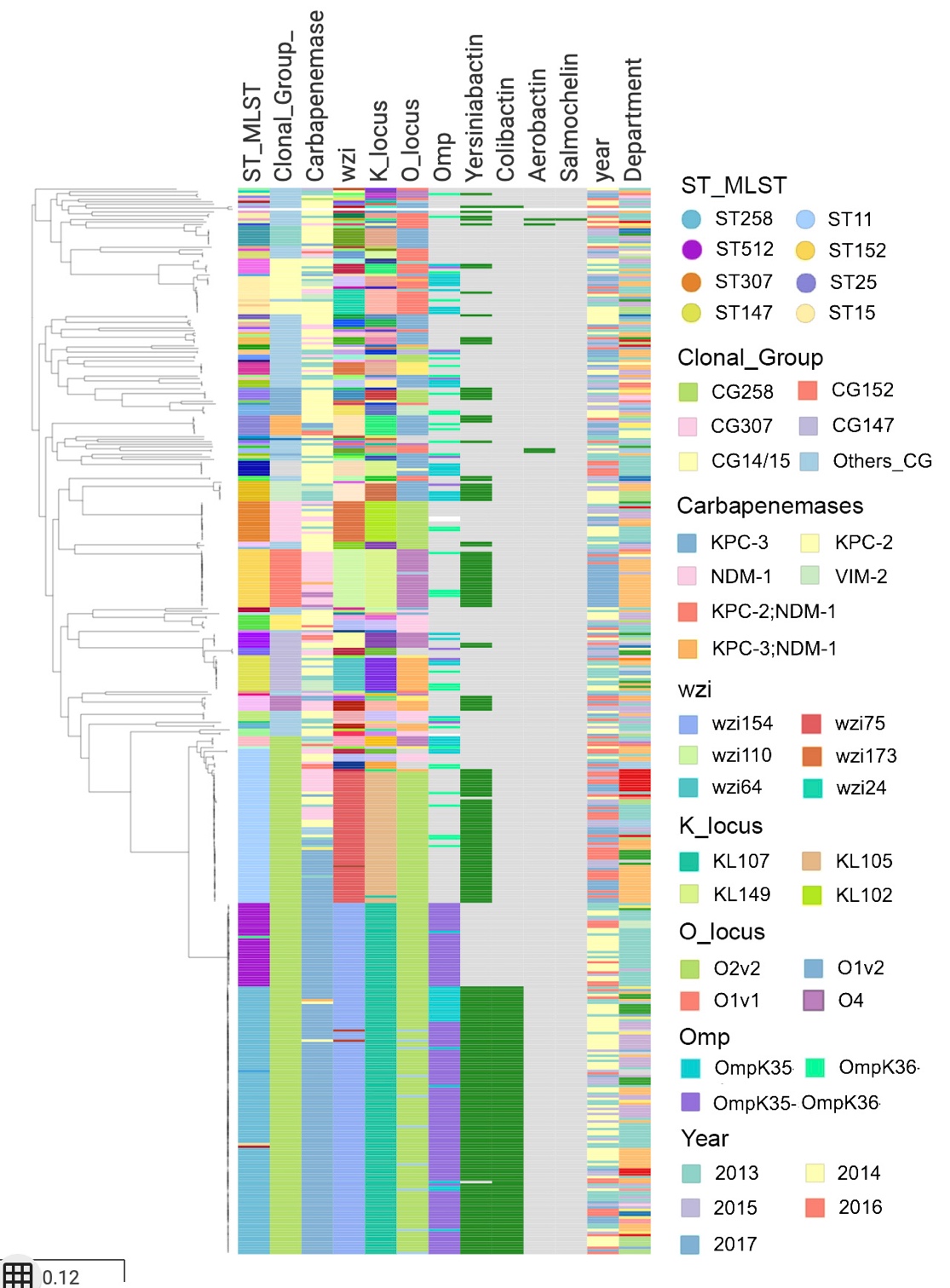


**Supplementary Figure 4.** Microreact visualization of 425 Colombian isolates of carbapenem-resistant *K. pneumoniae*. See visualizations on Microreact at: <https://microreact.org/project/vcsgT8Ic4/32d63ab7>.

**REFERENCES**

1. Ovalle M V, Saavedra SY, González MN, Hidalgo AM, Duarte C, Beltrán M. Results of the national surveillance of antimicrobial resistance of Enterobacteriaceae and Gram negative bacilli in health care-associated infections in Colombia, 2012-2014. *Biomedica* **2017**; 37: 473–485. Available at: http://dx.doi.org/10.7705/biomedica.v34i2.3432.
